# Antihyperlipidemic Effect, Identification and Isolation of the Lipophilic Components from *Artemisia integrifolia*

**DOI:** 10.3390/molecules24040725

**Published:** 2019-02-17

**Authors:** Yanhua Xu, Qinghu Wang, Wenqiang Bao, Biligetu Pa

**Affiliations:** College of Traditional Mongolian Medicine, Inner Mongolia Uaniversity for Nationalities, Tongliao 028000, China; baiyitianshixu@163.com (Y.X.); 19970101@163.com (W.B.); m15334964786@163.com (B.P.)

**Keywords:** *Artemisia integrifolia*, hyperlipidemia, lipophilic components, bioassay-guided fractionation

## Abstract

*Artemisia integrifolia* L. (Compositae) is a medicinal and edible plant. To investigate its antihyperlipidemic effect, a crude lipophilic extract and the composing compounds were isolated and fractioned from the petroleum ether extract of aerial parts of *A. integrifolia* using column chromatography on silica gel. The anti-hyperlipidemia effect was studied in a rat model of acute hyperlipidemia, which was induced by triton WR-1339. A new compound, integrinol (**4**), together with nine known compounds, namely chamazulene (**1**), acetylenes (E)-2 (**2**), acetylenes (E)-3 (**3**), eugenol (**5**), palmitic acid (**6**), oleic acid (**7**), linoleic acid (**8**), linolenic acid (**9**) and 12,13-epoxylinolenic acid were isolated from the crude lipophilic extract of *A. integrifolia*. The LD50 value of the crude extract was more than 4 g/kg. In Triton WR-1339-induced acute hyperlipidemia model, the crude lipophilic extract (200 mg/kg) significantly reduced total cholesterol (TC) by 70% (*p* ≤ 0.01) and triglycerides (TGs) by 94% (*p* ≤ 0.001). The fractioned compounds, such as chamazulene (**1**), acetylene-2 (**2**), and linolenic acid (**9**), used at 4 mg/kg dose, also significantly decreased the concentrations of TC (32%, 33% and 64%, respectively) and TGs (48%, 33% and 93%, respectively). These compounds (i.e., chamazulene, acetylenes (E)-2, and linolenic acid) were considered to be responsible for the bioactive antihyperlipidemic effect. In conclusion, the crude lipid extract of *Artemisia integrifolia* L. could be used as a potential treatment to avert hyperlipidemia. Further studies to confirm these results in other models of hyperlipidemia (e.g., diet-induced obesity) are warranted.

## 1. Introduction

Hyperlipidemia is defined as an increase in plasma lipids, which has been considered to be an important factor in the occurrence and severity of atherosclerotic cardiovascular diseases [1,2,3]. Clinical trials have shown that the increase of TC and TG in plasma is related to the development of atherosclerosis [4]. In the past few years, finding new drugs that can reduce and regulate the levels of TC and TG in plasma had been a hot topic, leading to numerous reports on the significant activity of natural drugs [5]. Herbal medicines have been proven to be powerful treatments for a variety of human diseases, including cancer, atherosclerosis, ulcers, diabetes, kidney disease and liver disease [6]. As an important component of national medicine, traditional Mongolian medicine is a Chinese medicine system localized in Inner Mongolia, with a long history of use of plant-based medicines in the treatment of cardiovascular diseases.

*A. integrifolia* (Composite) is widely distributed in Inner Mongolia where it is used as a medicinal and edible plant [7,8]. Modern pharmacological studies have demonstrated that its ethanol extract possesses very good superoxide, hydroxyl and nitric oxide free radical scavenging activities and can inhibit lipid peroxidation [9].

With regard to its chemical constituents, fatty acids, acetylenes, phenylpropanoids and terpenoids were isolated from the ether extract of *A. integrifolia* in previous studies [10,11,12]. These compounds exhibited many pharmacological activities [13,14]. For example, eugenol protects the liver from damage by scavenging free radicals, increasing the activity of SOD, GSH-px and CAT, and reducing the synthesis of MDA. Linolenic acid can increase the levels of EPA and DHA in platelets and erythrocytes, and reduce the production of arachidonic acid. However, until recently, there are only very few reports available on the antihyperlipidemic activity, identification and isolation of the lipophilic components from *A. integrifolia* [10], all of which may have a significant impact on the development of novel therapeutic options to treat hyperlipidemia, and related conditions.

## 2. Results and Discussion

### 2.1. Structure Elucidation of Compounds ***1***–***10*** from the Crude Lipophilic Components

**Figure 1:**
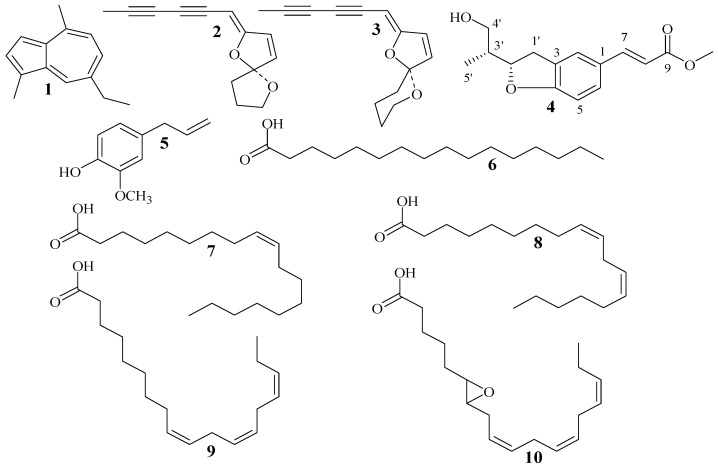
Structure of compounds **1**–**10**: chamazulene (**1**); acetylenes (E)-2 (**2**); acetylenes (E)-3 (**3**); integrinol (**4**); eugenol (**5**); palmitic acid (**6**); oleic acid (**7**); linoleic acid (**8**); linolenic acid (**9**); 12,13-epoxylinolenic acid (**10**).

For obtaining the most useful chemical information, column chromatography and nuclear magnetic resonance spectroscopy were used to isolate and identify the crude lipophilic components of *A. integrifolia*. A new compound, integrinol (**4**), was isolated and identified from crude lipophilic components by chromatographic fractionation, together with nine known compounds, namely chamazulene (**1**) [15], acetylenes (E)-2 (**2**) [16], acetylenes (E)-3 (**3**) [16], eugenol (**5**) [17], palmitic acid (**6**) [17], oleic acid (**7**) [17], linoleic acid (**8**) [18], linolenic acid (**9**) [19] and 12,13-epoxylinolenic acid (**10**) [19] (Figure 1). The purity of each compound was determined to be above 98% by normalization of the peak areas detected by HPLC.

Compound **4** was obtained as a white needles. UV (MeOH) λ_max_ (nm) (log *ε*): 263 (3.21); IR (KBr) *ν*_max_ (cm^−1^): 1685, 1653, 1585, 1481, 1308 and 1113 cm^−1^. The molecular formula was determined to be C_15_H_18_O_4_ by HR-ESI-MS (*m*/*z* 261.2920 [M − H]^−^; calcd. for 261.2931). The ^1^H-NMR spectrum (Figures S1–S6) of **4** (Table 1) showed two olefinic signals at δ_H_ 7.57 (1H, d, *J* = 15.5 Hz) and 6.42 (1H, d, *J* = 15.5 Hz), which suggested the presence of a *trans* olefinic group in compound **4**. In addition, the signals of three aromatic protons at δ_H_ 7.43 (1H, brd, *J* = 8.5 Hz), 6.77 (1H, d, *J* = 8.5 Hz), 7.39 (1H, brs) indicated the presence of an ABC system, which was confirmed by the HMBC correlations from δ_H_ 7.43 to δ_C_ 145.3 (C-7), 162.1 (C-4) and 125.2 (C-2), and 7.39 to δ_C_ 162.1 (C-4), 130.4 (C-6), 145.3 (C-7) and 31.6 (C-1′), and 6.77 to δ_C_ 126.9 (C-1) and 129.0 (C-3). The ^1^H-NMR of **4** also exhibited the presence of methyl protons at δ_H_ 0.86 (3H, s) and methoxy protons at δ_H_ 3.69 (3H, s).

In the HMBC spectrum (Table 1), the methoxy protons (δ_H_ 3.69) were correlated with the carbon signal at δ_C_ 167.5 (C-9), which revealed that the methoxy group was linked to C-9. Likewise, the methyl protons (δ_H_ 0.86) were correlated with the carbon signals at δ_C_ 85.7 (C-2′), 40.7 (C-3′) and 62.8 (C-4′). In addition, the HMBC correlations from δ_H_ 7.57 (1H, d, *J* = 15.5 Hz, H-7) to C-2 (δ_C_ 125.2), C-6 (δ_C_ 130.4), C-8 (δ_C_ 114.5) and C-9 (δ_C_ 167.5), and 6.42 (1H, d, *J* = 15.5 Hz, H-8) to C-1 (δ_C_ 126.9) and C-9 (δ_C_ 167.5), and 4.77 (1H, dd, *J* = 9.0, 4.5 Hz, H-2′) to C-1′ (δ_C_ 31.6), C-4′ (δ_C_ 62.8) and C-5′ (δ_C_ 12.3) further confirmed the structure of **4**. In the NMR spectrum, the coupling constant value, *J*_2′,3′_ = 4.5 Hz, suggested a *cis*-diaxial coupling between H-2′ and H-3′. In the NOESY spectrum, the H-2′ showed a strong correlation with H-3′, whereas the H-2′ showed no correlation with the proton at δ_H_ 1.01 (H-5′). If H-2′ and H-3′ adopt an *β*-orientation, H-5′ should be in an *α*-orientation [20]. Thus, the structure of compound **4** was elucidated and it was named integrinol.

### 2.2. Acute Toxicity

No mortality was observed in groups of rats treated with the crude lipophilic components. LD_50_ value for the crude lipophilic components was more than 4 g/kg.

### 2.3. Effects of Crude Lipophilic Components and Compounds ***1,2,9*** on Triton WR-1339-Induced Hyperlipidemia in Rats

Hyperlipidemia is one of the main causes of serious harm to human health. The incidence of hyperlipidemia in the general population was 20–40%, increasing at an estimated rate of ten thousand people per day [21]. High levels of TC, TG and LDL-C are the major risk factors for vascular disease, and high levels of HDL-C have a protective effect against its development. At present, although synthetics hypolipidemic drugs dominate the market, the toxic components of these drugs cannot be eliminated [22]. Herbal medicines are being studied and applied more to maintain our basic health because of the adverse effects of synthetic drugs and chemicals [23]. Many Mongolian medicinal plants, such as *Choerospondias axillaries* (Roxb.) Burtt et Hill [24], *Syringa pinnatifolia* Hemsl [25] and *Salvia miltiorrhiza* Bge [26], have been used to treat cardiovascular diseases.

The aim of this study was to investigate the hypolipidemic properties of the lipophilic components and some compounds isolated from *Artemisia integrifolia* in a model of hyperlipidemia induced by Triton WR-1339 in rats. Triton WR-1339 is a non-ionic detergent widely used to prevent the uptake of lipoproteins from plasma by peripheral tissues resulting in an increase in the levels of circulating lipid [27]. Triton-induced hyperlipidemia rat models are often used to screen natural or chemical hypolipidemic drugs [28]. Several studies have shown that systemic administration of Triton WR-1339 can rapidly increase plasma lipid levels in rats and mice [29].

Repeated daily oral treatments of rats with simvastatin (4 mg/kg), the crude lipophilic components (200 mg/kg) and compound **9** (4 mg/kg), consecutively, resulted in significant (*p* < 0.05) and dose-dependent decreases in the average body weight when compared to the untreated normal rats (Table 2). The TC, TGs, HDL-C and LDL-C levels in plasma of all groups 24 h after Triton WR-1339 injection are shown in Table 3.

Compared to the CG (control group), Triton WR-1339 caused a significant increase the levels of TC, TGs and LDL-C in plasma (*p* < 0.001) of the HG (hyperlipidemic group). In reality, the increase of TC level in plasma of the HG was 367% after 24 h in comparison with the CG. The TGs level in the HG was also elevated by 1640% after 24 h. Meanwhile, the LDL-C level in HG was increased by 252% after 24 h as compared to the CG, while a significant decrease (31%, *p* < 0.001) in the HDL-C was shown 24 h after Triton WR-1339 injection. Compared to the HG, the crude lipophilic components displayed a significant decrease in TC (35 and 70% at 100 and 200 mg/kg) and in TG (90 and 94% at 100 and 200 mg/kg); LDL-C level was reduced by 26 and 42% at 100 and 200 mg/kg, respectively; a significant increase in HDL-C by 41 and 56% at 100 and 200 mg/kg, respectively, was also indicated. Significantly elevated plasma TGs levels (by 87%, 48%, 33% and 93%) were exhibited in simvastatin-, compound **1**-, **2**- and **9**-treated rats, respectively, with respect to HG and in TC (69%, 32%, 33% and 64% in simvastatin-, compound **1**, **2** and **9**-treated rats). LDL-C level was reduced by 32%, 24%, 29% and 39% in simvastatin-, compound **1**, **2**- and **9**-treated rats, respectively; a significant increase in HDL-C by 20%, 53%, 72% and 31% in simvastatin-, compound **1**, **2**- and **9**-treated rats, respectively, was also observed.

The data in this study showed that the plasma TC, TGs and LDL-C levels were significantly decreased and the plasma HDL-C level was significantly increased at the 24 h of injection of Triton WR-1339 after the treatment with the crude lipophilic components in a dose-dependent manner for 15 d. The hypolipidemic action of the crude lipophilic components was obviously higher for TG than for TC. The results showed that oral dose of 4 mg/kg of chamazulene (**1**), acetylene (E)-2 (**2**) and linolenic acid (**9**) had antihyperlipidemic effect, because they could inhibit the elevation of TC, LDL-C and TG levels in Triton WR-1339-induced hyperlipidemia rat model. Compounds **1**, **2** and **9**- had similar hypolipidemic effects with crude lipophilic components, and their effects on TG were higher than those of TC. These results can be explained by understanding that the significant increase in plasma TC and TG levels after Triton WR-1339 administration is mainly due to the increased secretion of very low density lipoprotein (VLDL) by the liver. The proportion of TG in VLDL is several times that of cholesterol and decreases sharply with the decomposition and metabolism of VLDL and LDL [30]. After administration of Triton WR-133924 h, the level of HDL-C increased significantly with compounds **1**, **2** and **9**, which may have anti-atherosclerotic effects [31]. HDL enhanced the mobilization of TG and TC from plasma to liver and made them decompose and secrete in the form of bile acids [32].

In conclusion, the crude lipophilic components and compounds **1**, **2** and **9** could ameliorate the lipid profile in Triton-induced hyperlipidemic rats. The results of this study show a promising application prospect, but more studies are needed to understand the exact mechanism of these novel compounds as antihyperlipidemic agents and to clarify their structure-activity relationships.

## 3. Experimental Section

### 3.1. General Information

A Shimadzu UV-2201 spectrometer (Shimadzu, kyoto, Japan) was used to record the he UV spectra. The IR spectra were recorded in KBr discs on a Thermo Nicolet 200 double beam spectrophotometer (Shimadzu). A Waters Xevo G2-S QT (Waters, Milford, MA, USA) was used to measure the HR-ESI-MS spectra. NMR spectra were measured on a Bruker AV–500 spectrometer (Bruker, Karlsruhe, Germany) with tetramethylsilane (TMS) as the internal reference, and chemical shifts are expressed in ä (ppm). Column chromatography was performed by using silica gel (200–300 mesh, Marine Chemical Factory, Qingdao, China). Fractions were monitored by TLC (silica gel GF_254_10–40μm, Marine Chemical Factory, Qingdao, China), and spots were visualized by heating silica gel plates sprayed with 10% H_2_SO_4_ in EtOH.

### 3.2. Plant Material and the Crude Lipophilic Components from A. integrifolia

Aerial parts of *A. integrifolia* were collected in Humeng, Inner Mongolia (China), in June 2016. The plant materials were identified by Prof. Wuxiangjie (Inner Mongolia University for Nationalities) and voucher specimens (no. 20160628) were stored in the Mongolian Medicine Research Center, Inner Mongolia University for Nationalities. The powdered air dried aerial parts of *A. integrifolia* (5 kg) were extracted twice with petroleum (20 L) under reflux and concentrated under vacuum. The petroleum ether extract was fractionated by column chromatography on silica gel with hexane and hexane–ethyl acetate (*v*:*v*, 40:1). The hexane-ethyl acetate (*v*:*v*, 40:1) fraction was vacuum evaporated to recover the solvent giving the crude lipophilic components (900 g). The crude lipophilic components were stored in a refrigerator (0–4 °C) for further use.

### 3.3. Isolation of the Crude Lipophilic Components

The crude lipophilic components (50.0 g) was isolated by column chromatography on silica gel using a gradient of cyclohexane–ethyl acetate (80:1 to 10:1) to give 5 fractions (Fr. C_1–5_). Fr. C_1_ [(225 mg, cyclohexane–ethyl acetate (80:1) eluate] was chromatographed on silica gel column eluting with cyclohexane to give **1** (38 mg). Fr. C_2_ [(880 mg, cyclohexane–ethyl acetate (60:1) eluate] was subjected to silica gel column chromatography using cyclohexane–ethyl acetate with increasing polarity (80:1; 60:1; 40:1) to give **2** (49 mg), **3** (15 mg) and **4** (19 mg). Fr. C_3_ [(650 mg, cyclohexane–ethyl acetate (50:1) eluate] was separated by thin-layer chromatography (TLC) (cyclohexane–ethyl acetate, 20:1) yielding **5** (21 mg) and **6** (28 mg). Fr. C_4_ [(475 mg, cyclohexane–ethyl acetate (40:1) eluate] was separated by TLC (cyclohexane–ethyl acetate, 15:1) yielding **7** (19 mg) and **8** (25 mg). Fr. C_5_ [(320 mg, cyclohexane–ethyl acetate (20:1) eluate] was separated by TLC (cyclohexane–ethyl acetate, 10:1) yielding **9** (41 mg) and **10** (18 mg).

### 3.4. Characterization Data of Known Compounds

Chamazulene (**1**): Dark blue oil. ^1^H-NMR (CDCl_3_, 500 MHz) δ_H_: 8.17 (s, H-4, 1H), 7.64 (d, *J* = 3.0 Hz, H-2, 1H), 7.40 (d, *J* = 9.0 Hz, H-6, 1H,), 7.25 (d, *J* = 3.0 Hz, H-1, 1H), 7.03 (d, *J* = 9.0 Hz, H-7, 1H), 2.89 (q, *J* = 7.0 Hz, H-12, 2H), 2.83 (s, H-14, 3H), 2.66 (s, H-11, 3H), 1.39 (d, *J* = 7.0 Hz, H-13, 3H); ^13^C-NMR (CDCl_3_, 125 MHz) δ_C_: 144.4 (C-9), 136.6 (C-6), 136.0 (C-2), 135.6 (C-5), 134.5 (C-4), 127.1 (C-8), 125.5 (C-10), 125.3 (C-3), 124.8 (C-7), 112.5 (C-1), 33.6 (C-12), 24.4 (C-14), 12.6 (C-11), 17.7 (C-13).

Acetylene-2 (**2**): Yellowish oil. ^1^H-NMR (CDCl_3_, 500 MHz) δ_H_: 6.69 (d, *J* = 6.5 Hz, H-8, 1H), 6.22 (dd, *J* = 6.5, 2.0 Hz, H-9, 1H), 4.96 (s, H-6, 1H), 4.13–3.85 (m, H-13, 2H), 2.08–1.64 (m, H-11, 12, 4H), 1.95 (s, H-1, 3H); ^13^C-NMR (CDCl_3_, 125 MHz) δ_C_: 168.2 (C-7), 136.5 (C-9), 124.9 (C-8), 119.9 (C-10), 79.8 (C-6), 79.4 (C-2), 76.6 (C-4), 71.3 (C-5), 69.3 (C-13), 65.5 (C-3), 35.9 (C-11), 24.1 (C-12), 4.64 (C-1).

Acetylene-3 (**3**): Yellowish oil. ^1^H-NMR (CDCl_3_, 500 MHz) δ_H_: 6.67 (d, *J* = 6.5 Hz, H-8, 1H,), 6.25 (dd, *J* = 6.5, 1.0 Hz, H-9, 1H), 4.91 (s, H-6, 1H), 4.01 (td, *J* = 11.0, 3.0 Hz, Ha-14, 1H), 3.85 (1H, dd, *J* = 11.5, 4.0 Hz, Hb-14), 2.01 (s, H-1, 3H,), 1.87–1.59 (m, H-11, 12, 13, 6H); ^13^C-NMR (CDCl_3_, 125 MHz) δ_C_: 169.3 (C-7), 138.1 (C-9), 125.4 (C-8), 112.2 (C-10), 79.7 (C-2), 79.2 (C-6), 76.5 (C-4), 71.7 (C-5), 65.1 (C-3), 64.1 (C-14), 32.3 (C-11), 24.6 (C-12), 19.5 (C-13),4.70 (C-1).

Eugenol (**5**): Colourless oil. ^1^H-NMR (CDCl_3_, 500 MHz) δ_H_: 6.88 (d, *J* = 8.0 Hz, H-5, 1H), 6.73 (d, *J* = 8.0 Hz, H-6, 1H), 6.69 (s, H-2, 1H), 5.96–5.93 (m, H-8, 1H), 5.13–5.10 (m, Ha-9, 1H), 5.05–5.03 (m, Hb-9, 1H), 3.88 (s, 3H), 3.37 (d, *J* = 8.0 Hz, H-7, 2H); ^13^C-NMR (CDCl_3_, 125 MHz) δ_C_: 131.9 (C-1), 111.1 (C-2), 146.4 (C-3), 144.2 (C-4), 114.2 (C-5), 121.2 (C-6), 39.9 (C-7), 137.8 (C-8), 115.5 (C-9), -OCH3 (55.9).

Palmitic Acid (**6**): White solid. ^1^H-NMR (CDCl_3_, 500 MHz) δ_H_: 2.39 (t, *J* = 7.5 Hz, H-2, 2H), 1.69–1.65 (m, H-3, 2H), 1.38–1.36 (m, H-15, 2H), 1.35–1.29 (m, H-4~13, 16H), 1.27–1.25 (m, H-14, 2H), 0.90 (t, *J* = 6.5 Hz, H-16, 3H). ^13^C-NMR (CDCl_3_, 125 MHz) δ_C_: 178.9 (C-1), 34.1 (C-2), 32.1 (C-14), 29.7 (C-6, 7, 8, 9, 10, 11), 29.4 (C-5, 12), 28.9 (C-4, 13), 24.6 (C-3), 22.3 (C-15), 14.4 (C-16).

Oleic Acid (**7**) White solid. ^1^H-NMR (CDCl_3_, 500 MHz) δ_H_: 5.39 (d, *J* = 6.0 Hz, H-9, 1H), 5.29 (d, *J* = 6.0 Hz, H-9, 1H), 2.39 (t, *J* = 7.5 Hz, H-2, 2H), 2.05 (t, *J* = 7.5 Hz, H-8, 11, 4H), 1.66–1.63 (m, H-3, 2H), 1.35–1.33 (m, H-17, 2H), 1.32–1.28 (m, H-4~7, 12~15, 16H), 1.27–1.25 (m, H-16, 2H), 0.93 (t, *J* = 6.5 Hz, H-18, 3H). ^13^C-NMR (CDCl_3_, 125 MHz) δ_C_: 179.3 (C-1), 130.2 (C-10), 127.9 (C-9), 33.5 (C-2), 31.7 (C-16), 29.8 (C-5, 6, 13,14), 29.2 (C-7, 12), 29.0 (C-4, 15), 27.3 (C-8), 27.2 (C-11), 24.6 (C-3), 22.5 (C-17), 14.0 (C-18).

Linoleic Acid (**8**): White solid. ^1^H-NMR (CDCl_3_, 500 MHz) δ_H_: 5.45–5.31 (m, H-9, 10, 12, 13, 4H), 2.79 (t, *J* = 6.5 Hz, H-11, 2H), 2.41 (t, *J* = 7.5 Hz, H-2, 2H), 2.11 (t, *J* = 7.5 Hz, H-8, 14, 4H), 1.67–1.65 (m, H-3, 2H), 1.35–1.33 (m, H-17, 2H), 1.31–1.28 (m, H-4~7, 15, 10H), 1.26–1.24 (m, H-16, 2H), 0.92 (t, *J* = 6.5 Hz, H-18, 3H). ^13^C-NMR (CDCl_3_, 125 MHz) δ_C_: 179.6 (C-1), 130.3 (C-10), 129.9 (C-13), 128.3 (C-9), 127.7 (C-12), 34.3 (C-2), 31.6 (C-16), 29.6 (C-7), 29.4 (C-15), 29.2 (C-5), 29.1 (C-6), 29.0 (C-4), 27.5 (C-8), 27.2 (C-14), 25.8 (C-11), 24.6 (C-3), 22.5 (C-17), 14.1 (C-18).

Linolenic Acid (**9**): White solid. ^1^H-NMR (CDCl_3_, 500 MHz) δ_H_: 5.45–5.33 (m, H-9, 10, 12, 13, 15.16, 6H), 2.85 (t, *J* = 6.5 Hz, H-11, 14, 4H), 2.37 (t, *J* = 7.5 Hz, H-2, 2H), 2.09 (q, *J* = 7.5 Hz, H-17, 2H), 2.06–2.04 (m, H-8, 2H), 1.63–1.60 (m, H-3, 2H), 1.35–1.31 (m, H-4~7, 8H), 0.96 (t, *J* = 7.5 Hz, H-18, 3H). ^13^C-NMR (CDCl_3_, 125 MHz) δ_C_: 131.9 (C-16), 130.5 (C-9), 128.1 (C-12), 128.4 (C-13), 127.9 (C-10), 127.1 (C-15), 80.5 (C-1), 34.1 (C-2), 29.6 (C-6), 29.3 (C-5), 29.1 (C-4, 7), 27.7 (C-8), 25.7 (C-11), 25.5 (C-14), 24.7 (C-3), 20.5 (C-17), 14.1 (C-18).

12,13-Epoxylinolenic Acid (**10**): White solid. ^1^H-NMR (CDCl_3_, 500 MHz) δ_H_: 5.39–5.34 (m, H-9, 10, 12, 13, 15,16, 6H), 4.31–4.29 (m, H-7, 1H), 4.27–4.26 (m, H-6, 1H), 2.34 (t, *J* = 7.5 Hz, H-2, 2H), 2.79 (t, *J* = 6.5 Hz, H-11, 14, 4H), 2.09–2.07 (m, H-8, 2H), 2.05 (q, *J* = 7.5 Hz, H-17, 2H), 2.03–2.01 (m, H-5, 2H), 1.63–1.61 (m, H-3, 2H), 1.35–1.33 (m, H-4, 2H), 0.90 (t, *J* = 7.5 Hz, H-18, 3H). ^13^C-NMR (CDCl_3_, 125 MHz,) δ_C_: 173.5 (C-1), 131.7 (C-16), 130.2 (C-9), 128.5 (C-13), 128.2 (C-12), 127.9 (C-10), 127.3 (C-15), 68.8 (C-7), 62.2 (C-6), 34.1 (C-2), 29.3 (C-4), 27.4 (C-5), 25.8 (C-11, 14), 25.5 (C-8), 24.8 (C-3), 20.3 (C-17), 14.1 (C-18).

### 3.5. Experimental Animals

Male Wistar rats weighing 180–200 g were provided by Changchun Yisheng Laboratory Animal Technology Co., Ltd. (Changchun, China). The rats were kept in polypropylene cages under standard animal feeding conditions (25 ± 5 °C, 40–70% RH, 12 h light/dark cycle). Standard pellet feeds [Sterile pellet feeds for rats, product executive standard: GB14924–2001, license: SCXK-(-JI-) 2010-0001] and water were obtained with free access.

### 3.6. Acute Toxicity

For the evaluation of acute toxicity, male Wistar rats weighing 180–200 g were divided into groups of 10 animals. The crude lipophilic components were given by intra-gastric administration at the doses of 1, 2 and 4 g/kg from first to third groups, respectively [33]. The control group received an intra-gastric administration of corn oil (5 mL/kg). The mortality rate within 72 h period was observed and the LD_50_ was estimated on the basis of the method described by Silva et al. [34].

### 3.7. Triton WR-1339-Induced Hyperlipidemic in Rats

Overnight fasted rats (64) were randomly divided into eight groups each consisting of eight animals. The rats were also treated with daily oral treatment for 15 d before treatments with intraperitoneal injection of triton WR-1339 (200 mg/kg) once as follows: Group I served as control group (CG) receiving an intraperitoneal injection of normal saline and an intra-gastric administration of corn oil. Group II served as hyperlipidemic group (HG) receiving Triton WR-1339 (i.p.) dissolved in normal saline and an intra-gastric administration of corn oil. Group III (positive control group, PCG) was treated Triton WR-1339 (i.p.) and an intra-gastric administration of simvastatin (4 mg/kg). Group IV–VIII (test groups, TG IV–VIII) received Triton WR-1339 (i.p.) and intra-gastric administration of the crude lipophilic components (100 and 200 mg/kg) and compounds **1**, **2** and **9** (4 mg/kg) dissolved in corn oil, respectively. In the course of the 15 d oral treatment, body weights of rats were regularly taken from 1st to 15th day, respectively. Blood samples were collected from abdominal aorta after 24 h of injection of Triton WR-1339 in ethylenediaminetetraacetic acid (EDTA) containing tubes; plasma was separated and stored at −18 °C until determination [35].

### 3.8. Biochemical Analysis

TC, TGs, Low-density lipoprotein cholesterol (LDL-C) and High-density lipoprotein cholesterol (HDL-C) levels in the plasma were estimated by an enzymatic method with an automatic analyzer (Model XL-300, Erba, Mannheim, Germany) [36].

### 3.9. Statistical Analysis

The results were expressed as mean ± SD. Data obtained were analyzed using variance analysis and differences with *p* < 0.05 were considered statistically significant.

## 4. Conclusions

In summary, the observed characteristics clearly confirm the medicinal use of the plant in preventing hyperlipidemia. The lipophilic components and compounds **1**, **2** and **9** from *Artemisia integrifolia* have positive effects on plasma lipoprotein profiles, suggesting that they have good therapeutic potential in improving hyperlipidemia.

## Figures and Tables

**Table 1:** ^1^H and ^13^C-NMR data (500 and 125 MHz, resp.; *d*_6_-DMSO) of integrinol (**4**).

Position	δ_H_ (ppm), *J* (Hz)	δ_C_ (ppm)	HMBC
1	—	126.9	
2	7.39 (brs, 1H)	125.2	C-4, C-6, C-7, C-1′
3	—	129.0	
4	—	162.1	
5	6.77 (d, 1H, *J* = 8.5 Hz)	109.5	C-1, C-3
6	7.43 (brd, 1H, *J* = 8.5 Hz)	130.4	C-2, C-4, C-7
7	7.57 (d, 1H, *J* = 15.5 Hz)	145.3	C-2, C-6, C-8, C-9
8	6.42 (d, 1H, *J* = 15.5 Hz)	114.5	C-1, C-9
9	—	167.5	
1′	3.17 (dd, 1H, *J* = 16.0, 9.0 Hz) 2.98 (dd, 1H, *J* = 16.0, 8.5 Hz)	31.6	C-2, C-4, C-2′, C-3′
2′	4.77 (dd, 1H, *J* = 9.0, 4.5 Hz	85.7	C-3, C-4′, C-5′
3′	1.95 (m, 1H)	40.7	
4′	3.51 (dd, 1H, *J* = 10.5, 5.0 Hz) 3.05 (dd, 1H, *J* = 10.5, 6.5 Hz)	62.8	C-2, C-4, C-2′, C-3′
5′	0.86 (d, 3H, *J* = 7.0 Hz)	12.3	C-2′, C-3′, C-4′
-OCH3	3.69 (3H, s)	51.7	C-9

**Table 2:** Effect of repeated daily oral treatments with simvastatin, the crude lipophilic components and compounds in the average body weightof rats on day 1 and 15 before intraperitoneal injection of triton WR-1339.

Groups	Average Body Weight (g)	
	Day 1	Day 15
I	195.73 ± 9.77	250.15 ± 9.96
II	195.12 ± 11.73	249.63 ± 12.86
III	195.91 ± 11.07	233.75 ± 6.59 *
IV	198.06 ± 10.77	240.85 ± 4.55
V	196.7 ± 11.29	231.28 ± 9.89 *
VI	198.41 ± 10.86	242.67 ± 10.83
VII	199.35 ± 11.92	247.55 ± 10.50
VIII	198.93 ± 11.96	232.08 ± 11.71 *

* Represent significant decreases at *p* < 0.05, when compared to Group I values.

**Table 3:** Effect of the crude lipophilic components and compounds **1**, **2**, **9** on plasma lipid levels in Triton WR-1339-induced hyperlipemic rats after 24 h.

Parameters	TGs (mmol/L)	TC (mmol/L)	HDL-C (mmol/L)	LDL-C (mmol/L)
CG	0.68 ± 0.15	1.53 ± 0.18	1.17 ± 0.19	0.27 ± 0.07
HG	11.8 ± 1.32 ^###^	7.14 ± 1.73 ^###^	0.81 ± 0.10 ^###^	0.95 ± 0.21 ^###^
PCG (simvastatin, 4 mg/kg)	1.53 ± 1.04 **	2.19 ± 0.64 **	0.97 ± 0.25	0.65 ± 0.15 **
TG IV (crude lipophilic components, 100 mg/kg)	1.19 ± 0.56 ***	4.65 ± 1.40 *	1.15 ± 0.15 *	0.70 ± 0.23 *
TG V (crude lipophilic components, 200 mg/kg)	0.69 ± 0.16 ***	2.13 ± 0.50 **	1.26 ± 0.26 **	0.55 ± 0.19 **
TG VI (compound **1**, 4 mg/kg)	6.13 ± 1.52 **	4.80 ± 1.67 *	1.24 ± 0.34 **	0.72 ± 0.20 *
TG VII (compound **2**, 4 mg/kg)	7.93 ± 1.41 **	4.77 ± 1.48 *	1.39 ± 0.52 **	0.67 ± 0.17 **
TG VIII (compound **9**, 4 mg/kg)	0.75 ± 0.22 ***	2.54 ± 0.75 ***	1.06 ± 0.12 *	0.58 ± 0.14 **

All values represent mean ± SEM for eight animals. ^###^ (*p* ≤ 0.001) Values deviate very significantly from the CG values. *** (*p* ≤ 0.001), ** (*p* ≤ 0.01) and *(*p* ≤ 0.05) Values deviate significantly from the HG values.

## Supplementary Materials

The following are available online at http://www.mdpi.com/1420-3049/24/4/725/s1. Figures S1–S6: 1D (1H NMR and ^13^C NMR) and 2D-NMR (COSY, HSQC HMBC and NOESY) spectra of integrinol (**4**).
