# Supplementary material for: Antihyperlipidemic Effect, Identification and Isolation of the Lipophilic Components from Artemisia integrifolia
Source: Molecules. 2019 Feb 17;24(4):725. doi: 10.3390/molecules24040725 (PMC6412335; doi:10.3390/molecules24040725)
Supplement: Supplementary file 1 [file molecules-24-00725-s001.pdf]

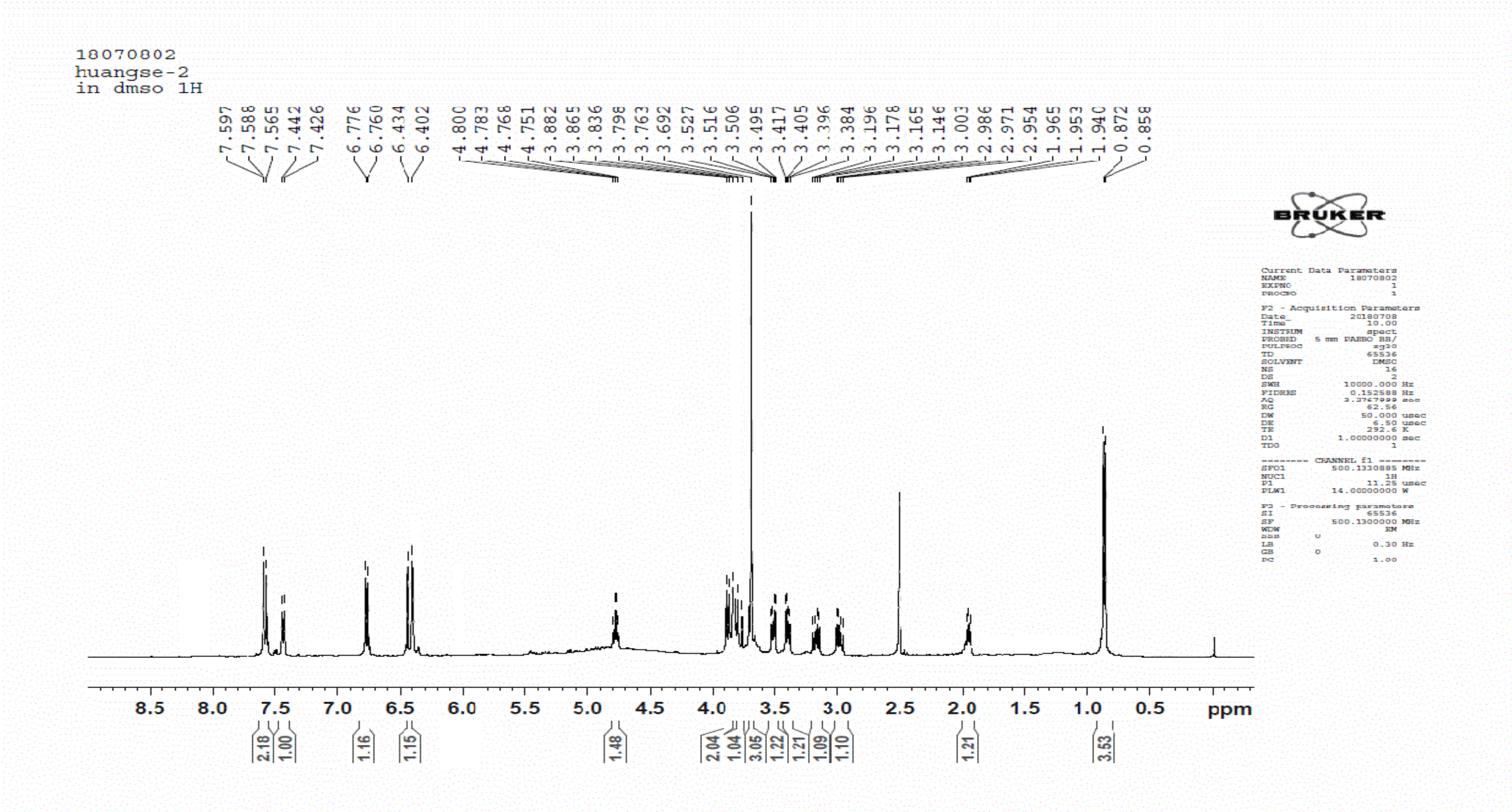

Figure S1. ^{1}H-NMR spectra of compound **4**

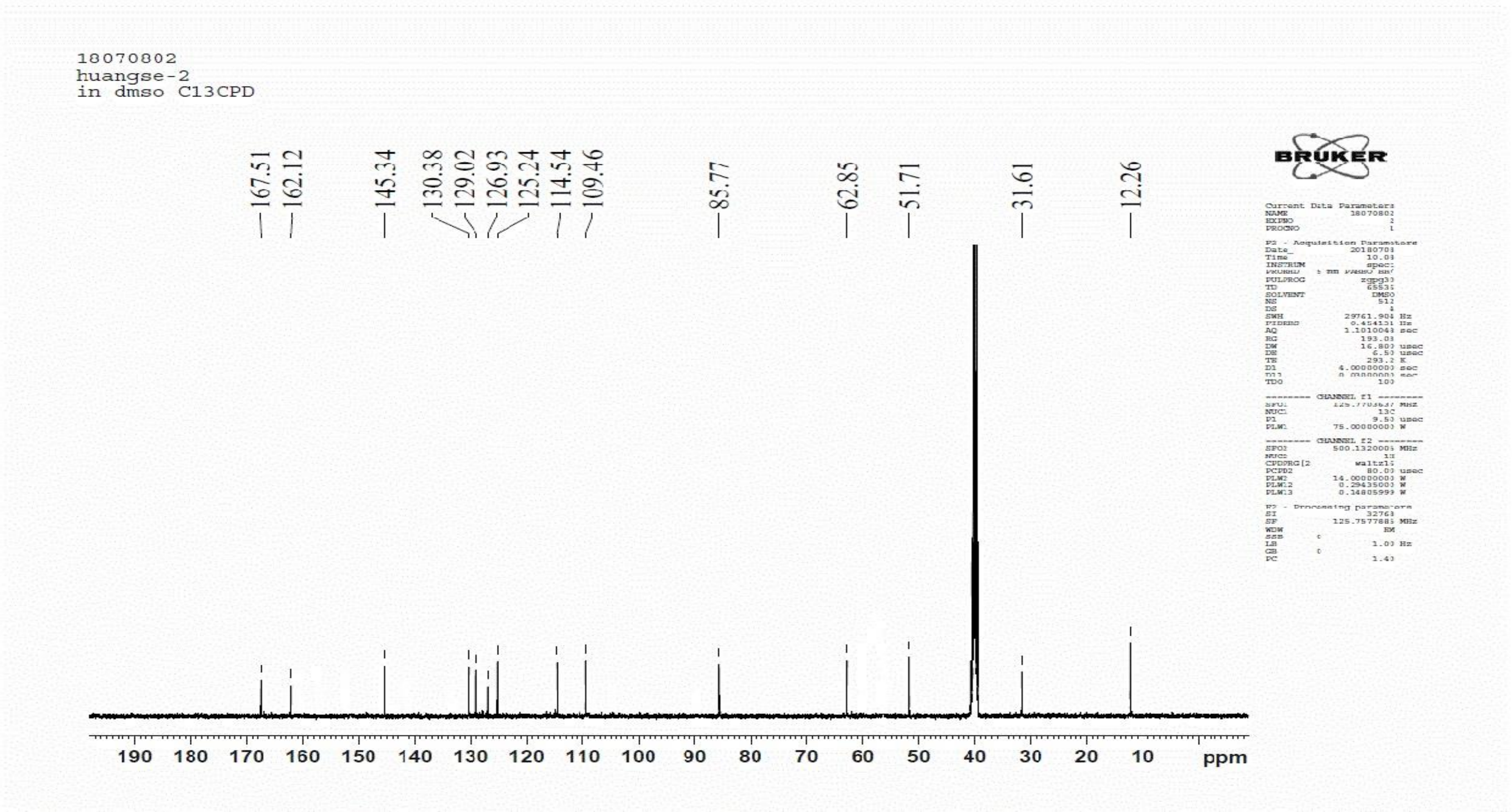

Figure S2. ^{13}C-NMR spectra of compound **4**

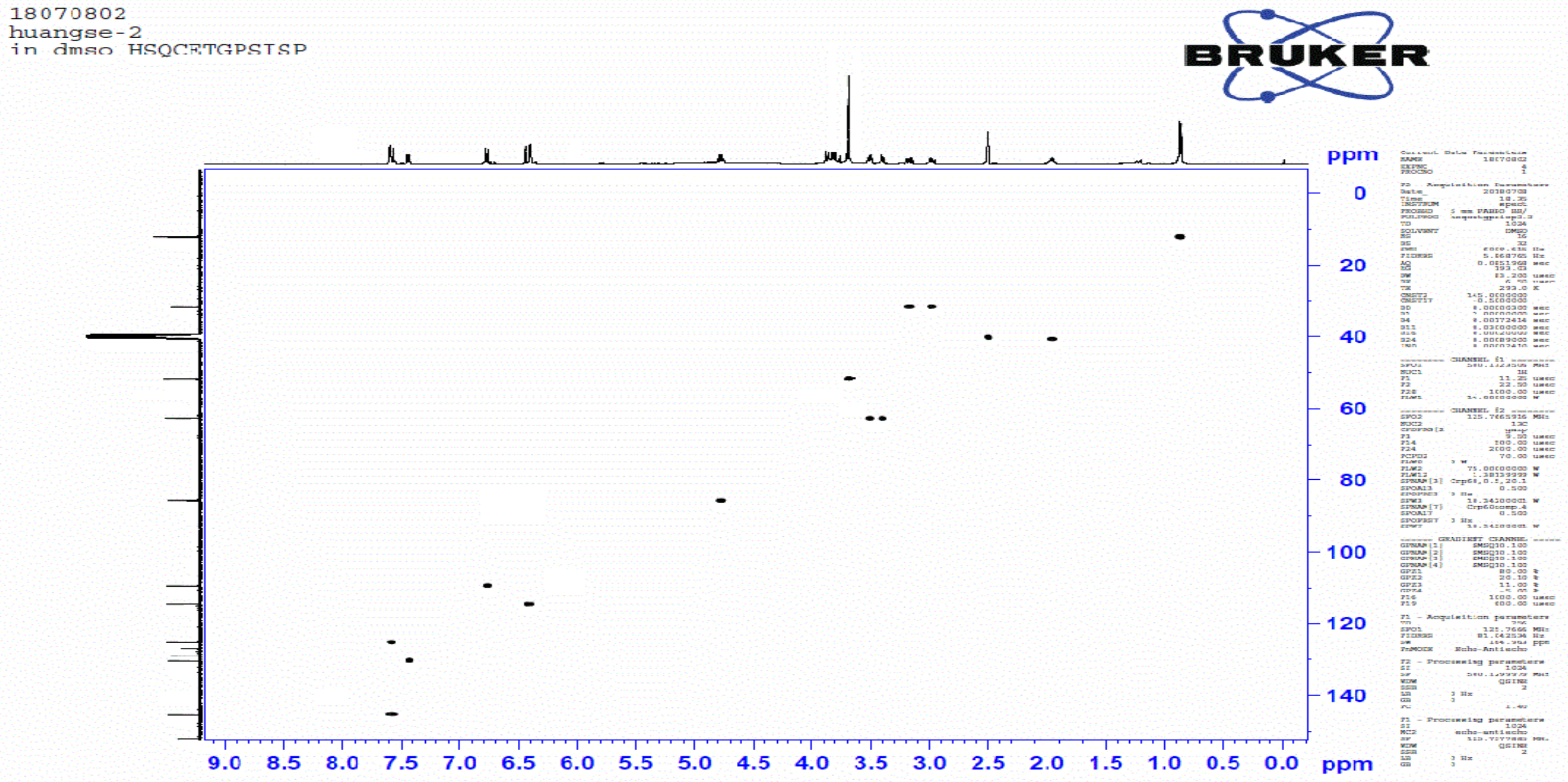

Figure S3. HSQC spectra of compound **4**

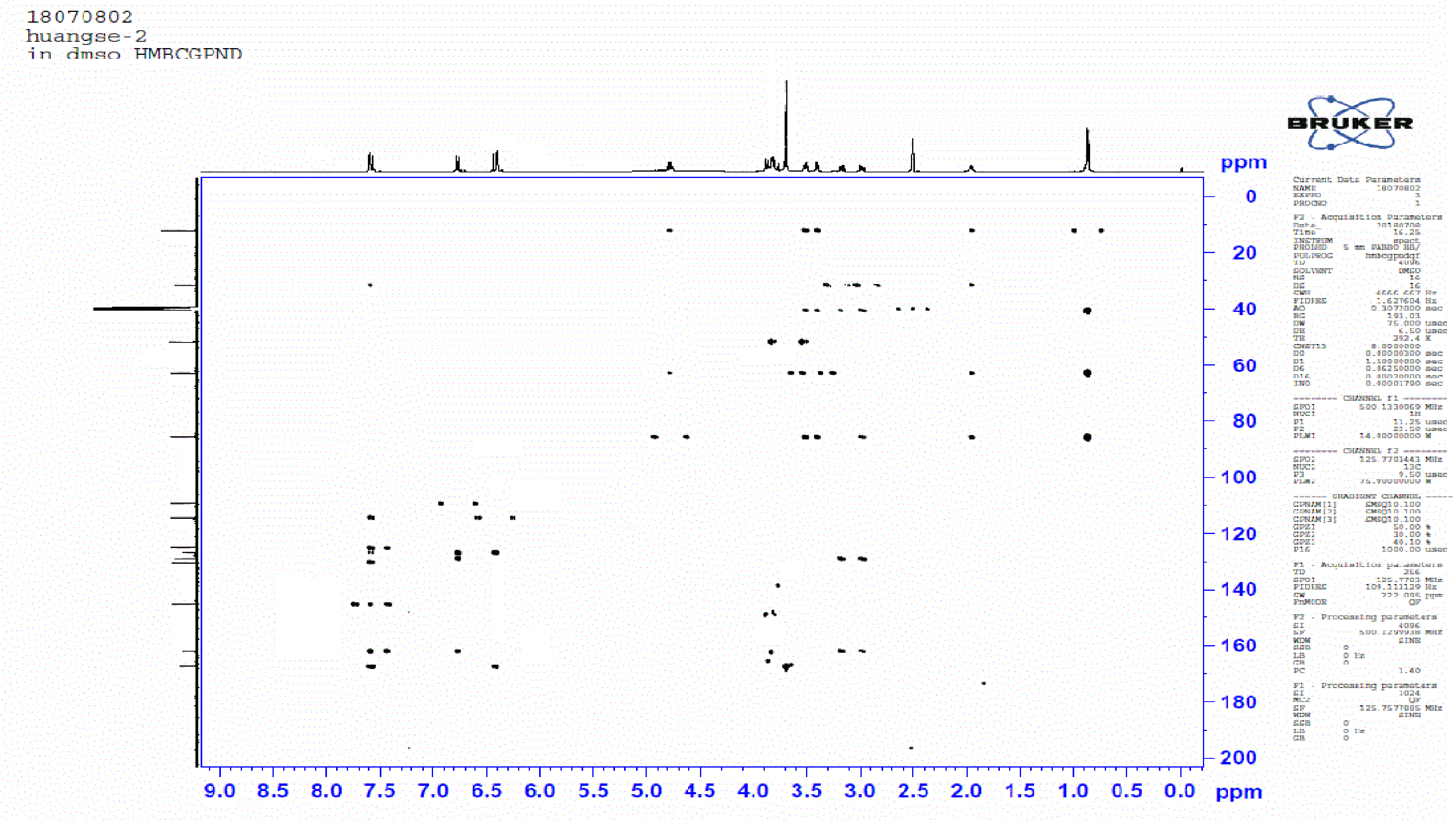

Figure S4. HMBC spectra of compound **4**

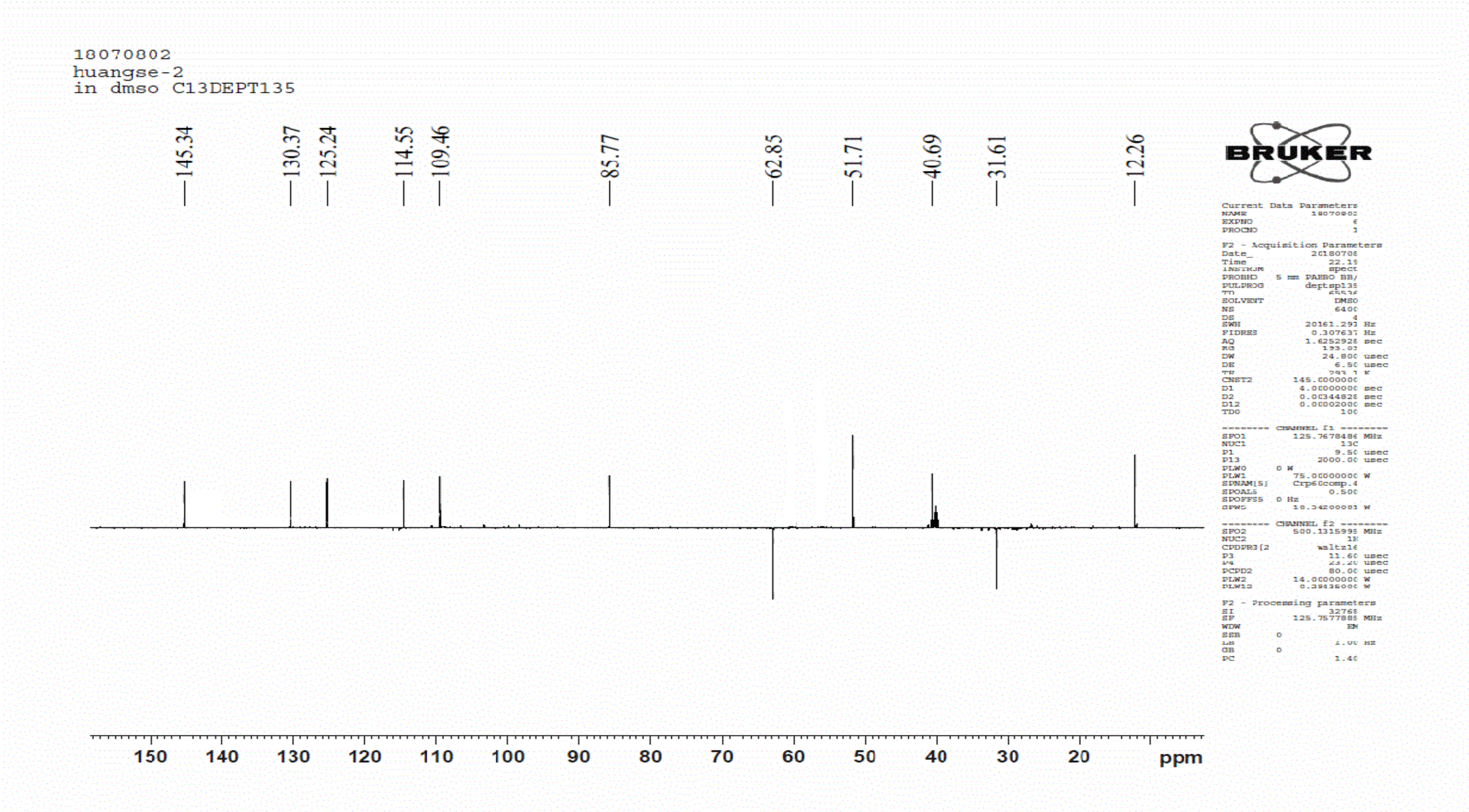

Fig, S5 DEPT spectrum of compound **4**

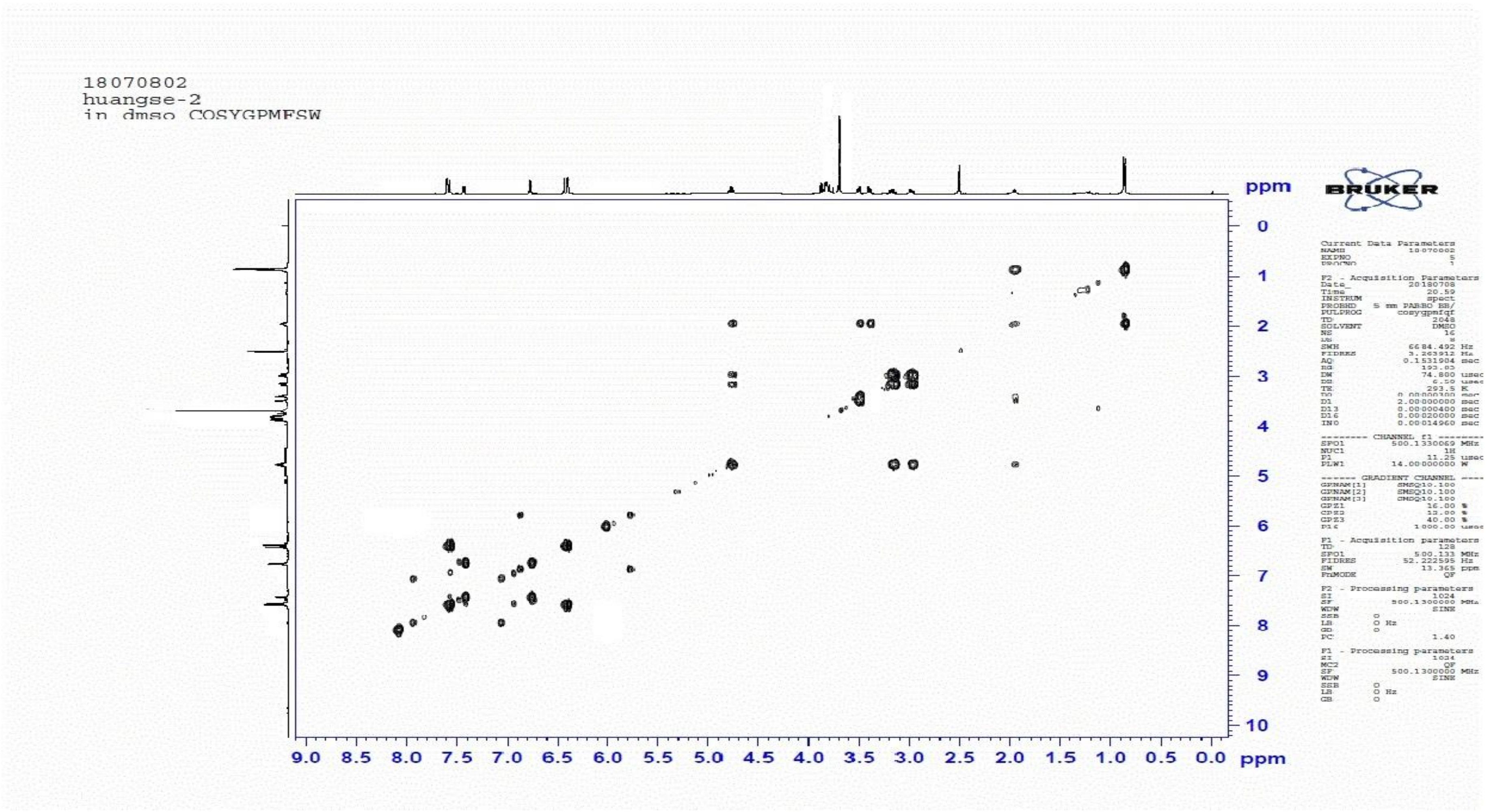

Fig. S6 COSY spectrum of compound **4**
